# Bacteria-archaea metabolic complementarity as a driver of ecosystem functioning in Chinese coastal sediments

**DOI:** 10.3389/fmicb.2026.1785657

**Published:** 2026-02-17

**Authors:** Xi Yuan, Xiao-Lin Liu, Si-Qi Ye, Shou-Qing Ni, Zhi-Bin Wang

**Affiliations:** 1 School of Life Sciences, Shandong University, Qingdao, Shandong, China; 2 Shenzhen Research Institute of Shandong University, Shenzhen, Guangdong, China; 3 Institute of Marine Science and Technology, Shandong University, Qingdao, Shandong, China; 4 School of Environmental Science and Engineering, Shandong University, Qingdao, Shandong, China

**Keywords:** archaea, bacteria, biogeochemical cycling, marine sediment, metabolic complementarity, methane cycling

## Abstract

**Introduction:**

The East China Sea (ECS), a continental shelf sea influenced by Yangtze River discharge and human activities, hosts highly diverse and structurally heterogeneous microbial assemblages in shallow sediments, shaped by complex hydrological and biogeochemical gradients.

**Methods:**

Here, 16S rRNA gene high-throughput sequencing and environmental parameter analysis across three depth intervals (50 m, 50–100 m, 100–200 m) were used to systematically characterize the vertical distribution of bacterial and archaeal communities.

**Results and discussion:**

Multivariate statistics identified organic carbon, oxygen, and sulfur availability as key drivers of microbial community structure. Co-occurrence network and functional profiling uncovered distinct ecological divergence: bacteria dominate oxidative processes including nitrogen and sulfur cycling as well as organic matter degradation, while archaea, predominantly *Bathyarchaeia*, occupy modular anaerobic niches specialized in methanogenesis and reductive pathways. This functional complementarity sustains integrated biogeochemical cycling in dynamic marine sediments. Our study advances understanding of prokaryotic community responses to vertical environmental gradients and their ecological roles in coastal sediment biogeochemical cycling.

## 1 Introduction

The bacterial and archaeal communities in coastal sediments serve as an invisible engine, maintaining the health and balance of this ecosystem. They collectively drive crucial biogeochemical cycles: Bacteria act as the primary force, efficiently decomposing organic matter and releasing nutrients for other organisms to utilize (Hicks et al., 2018). Archaea primarily govern the process of methane production (Wöhlbrand et al., 2025) and yet both groups work in synergy through anaerobic oxidation of methane (AOM), effectively reducing the release of greenhouse gasses (Song, 2010). Furthermore, they jointly regulate the transformation of elements such as sulfur and nitrogen. Particularly through denitrification and anaerobic ammonium oxidation (anammox), they act as a natural “biofilter,” removing excess nitrogen from the water body and preventing eutrophication disasters (Zhang et al., 2025). The complex activities of these microbes not only accomplish nutrient regeneration and pollutant purification but also have a profound impact on the productivity of the entire coastal ecosystem and global climate change (Hutchins and Fu, 2017).

However, although microbial assemblages in coastal sediments are widely recognized as key drivers of ecosystem functions, their specific functional traits and synergistic mechanisms in biogeochemical cycles remain poorly understood. Previous studies have largely focused on microbial diversity in surface waters and deep-sea environments, revealing that environmental factors such as temperature, salinity, and nutrient availability significantly influence community composition (Wright et al., 2012; Yang et al., 2024). Current research has largely been limited to descriptions of community structure, leaving significant knowledge gaps in the identification of functional potential, niche differentiation of key functional taxa, and their coupling with environmental factors (Fuhrman et al., 2015). How functional traits of sediment microbes mediate their niche differentiation under anthropogenic and climatic perturbations not only hinders research progress in this field, but also severely constrains the accurate prediction of coastal ecosystem responses to human activities and climate change.

The East China Sea (ECS) represents a highly typical and valuable system for studying microbial assemblages in coastal sediments, owing to its distinctive and representative environmental gradients (Liu et al., 2011). Its shelf region exhibits pronounced depth variations, extending from shallow nearshore waters to deeper offshore areas, resulting in significant changes in hydrostatic pressure, light penetration, and organic matter deposition (Lian et al., 2023). Furthermore, the complex circulation system, characterized by the interplay of the nutrient-rich Yangtze River diluted water, the warm and saline Kuroshio Current, and seasonal monsoon-driven currents, creates strong physicochemical gradients that shape sediment properties and microbial habitats (Maotian et al., 2007). The ECS is subject to substantial anthropogenic pressures, including eutrophication from agricultural and urban runoff, bottom trawling, and potential pollutant accumulation, which collectively influence sediment composition, nutrient availability, and microbial metabolic processes (Guo et al., 2019). These combined natural and human-induced factors make the ECS an ideal model system for investigating the composition, diversity, function, and ecological responses of sediment-associated microbial assemblages under multiple environmental stressors.

Therefore, this study systematically investigates the vertical changes in bacterial and archaeal community structures, key environmental driving factors, functional potential, and ecological co-occurrence patterns across multiple sediment sampling sites in the shallow ECS. By integrating high-throughput 16S rRNA sequencing and comprehensive environmental parameter profiling, we aim to uncover the vertical ecological adaptation strategies of prokaryotes in coastal sediment environments, and to provide a microbial perspective for understanding the mechanisms of marine carbon, nitrogen, and sulfur cycling under the influence of global change.

## 2 Materials and methods

### 2.1 Sample collection and environmental parameter measurement

**Figure 1:**
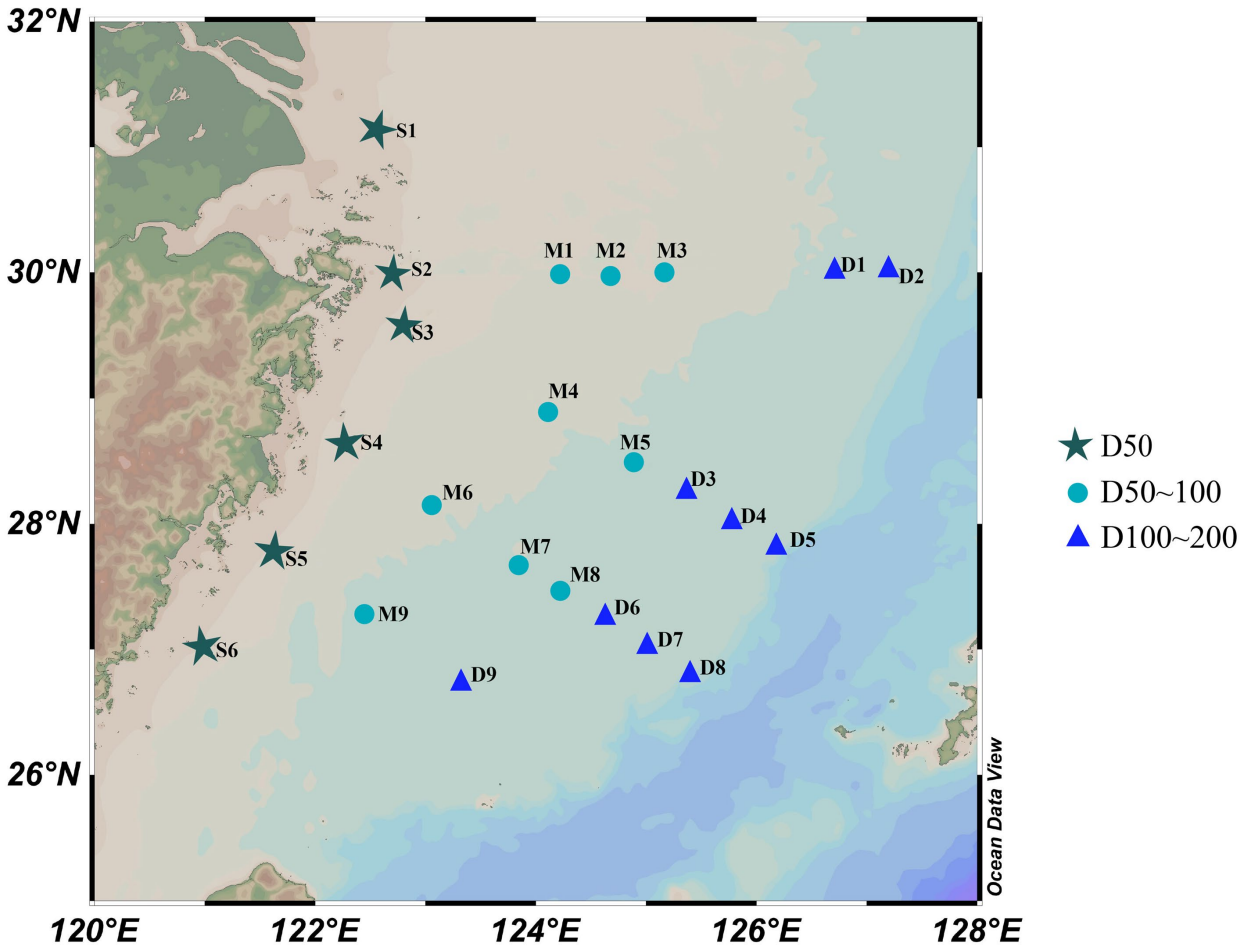
Sample collection sites at sediment depths of 50, 50–100, and 100–200 m.

In the spring of 2024, surface sediment samples were collected from 24 stations across the general area of ECS from latitude 26.76°N to 31.16°N and longitude 121°E to 127.2°E. The voyage number is NORC2024-02, covering a primary area that extends northward from the Yangtze River Estuary (the boundary between the Yellow Sea and the ECS), eastward to the western shelf of the Okinawa Trough, and southward to the northeastern waters of Taiwan. The stations are systematically distributed across three core ecological zones of the ECS: the coastal zone affected by terrestrial input, the shelf break zone with significant hydrological gradients, and the offshore shelf zone with a stable sedimentary environment. They are divided into three water depth groups: 50 m, 50–100 m, and 100–200 m (Figure 1), avoiding sampling bias caused by localized distribution. Sediments were extracted using PVC corers, evenly divided, and stored at −20 °C for subsequent DNA extraction and other analyses. The sediments were dried, homogenized, and sieved through a 2.0 mm mesh. From 5 g of the sieved sediment, nitrite (NO_2_^−^), ammonium (NH_4_^+^), and nitrate (NO_3_^−^) were extracted using 30 mL of 1 mol/L KCl with shaking for 1 h at room temperature, and measured using a UV–visible spectrophotometer (T6, Puxi General, China). Chlorophyll a was measured by spectrophotometry. The sediment samples were further finely ground and sieved (<0.15 mm), and 10 mg was weighed for total organic carbon (TOC), total sulfur (TS), and total nitrogen (TN) analysis using an elemental analyzer (UNICUBE, Elementar, Germany). Seawater properties, including temperature, depth, salinity, dissolved oxygen, conductivity, colored dissolved organic matter (CDOM), and pressure, were measured using a CTD instrument (Sea-Bird Scientific, United States).

### 2.2 Genomic DNA extraction and high-throughput sequencing

DNA was extracted from the sediment samples using the DNeasy® PowerSoil DNA Kit (QIAGEN), following the manufacturer’s instructions. DNA concentrations were quantified using a NanoDrop One spectrophotometer (Thermo Scientific, United States). The V3-V4 hypervariable regions of the bacterial 16S rRNA gene were amplified using primers 338F (ACTCCTACGGGAGGCAGCAG) and 806R (GGACTACHVGGGTWTCTAAT) (Behrendt et al., 2012), while the archaeal V4-V5 regions were amplified using primers 524F (TGYCAGCCGCCGCGGTAA) and 958R (YCCGGCGTTGAVT CCAATT) (Pires et al., 2012). PCR amplification was performed using an ABI GeneAmp® 9700 thermal cycler (ABI, United States), by amplifying the bacterial and archaeal 16S rRNA genes with two optimized PCR systems: the bacterial V3-V4 hypervariable regions were amplified using Pro Taq DNA polymerase, while the archaeal V4-V5 regions were amplified using the high-fidelity TransStart FastPfu DNA polymerase to ensure amplification accuracy, both in 20 μL reaction volumes. After amplification, PCR products were recovered using an AxyPrep DNA gel extraction kit (AXYGEN) via gel excision, followed by elution with Tris–HCl buffer. The products were then pooled and verified by 2% agarose gel electrophoresis. High-throughput sequencing was performed by Personal Biotechnology Co., Ltd. (Shanghai, China) on the Illumina NovaSeq PE250 platform with paired-end reads (2 × 250 bp). Sequencing data were subjected to quality control, denoising, and taxonomic annotation to generate amplicon sequence variants (ASVs). All sequencing services were provided by Shanghai Personalbio Technology Co., Ltd. The raw sequencing data of Bacteria and Archaea have been uploaded to the NCBI with project number of PRJNA1309059 and PRJNA1309283, respectively.

### 2.3 Microbial community diversity analysis

The annotated ASV data were then analyzed based on sampling locations and depth layers (Lawley and Tannock, 2017). Alpha diversity was assessed using several indices, including the Chao1 (Chao et al., 2014) and Observed ASV numbers indices for species richness, and the Shannon and Simpson indices to characterize community heterogeneity (Magurran, 2021). Non-metric multidimensional scaling (NMDS) was applied to the Bray–Curtis dissimilarity matrix to reduce dimensionality and visually illustrate structural differences between bacterial and archaeal communities (Clarke, 1993). Additionally, potential relationships between geographic distance and microbial community similarity (based on Bray-Curtis distance) were analyzed using R version 4.4.5. To further clarify the environmental drivers of community variation, canonical correspondence analysis (CCA) was employed to identify and quantify the key environmental parameters that significantly influenced the structural variability of bacterial and archaeal communities (ter Braak, 1986).

### 2.4 Analysis of environmental drivers

Community-environment association analyses were conducted based on ASV-level data and the top 30 most abundant genera. Specifically, Mantel tests were used to evaluate correlations between microbial community structure (based on Bray–Curtis dissimilarity matrices) and environmental gradients (based on Euclidean distance matrices) (Ramette, 2007). To control for multiple testing errors and ensure statistical robustness, all analyses were validated using 9,999 permutation tests and *p*-values were corrected using the false discovery rate (FDR) method. A significance threshold of *p* < 0.05 was applied (Anderson, 2001). Correlation strength was categorized based on the correlation coefficient (*r*) as follows: strong correlation (*r* ≥ 0.5), moderate correlation (0.25 ≤ *r* < 0.5), and weak correlation (*r* < 0.25) (Stegen et al., 2013). Finally, heatmaps were generated using the Chiplot platform^1^ to analyses differentiated responses of microbial assemblages at the ASV and genus levels to environmental heterogeneity (Gu et al., 2016). These visualizations uncovered how community variations were driven by environmental factors across different taxonomic resolutions. Additionally, scatter plots with regression lines were used to identify strong co-variation patterns among environmental parameters (Boldina and Beninger, 2016).

### 2.5 Community structure and molecular ecological network construction

At the genus level, the bacterial and archaeal community compositions in the East China Sea sediments were classified and analyzed. Molecular ecological networks were constructed using the Molecular Ecological Network Analysis Pipeline (MENA) platform, and visualized with Gephi 0.10. The intra-domain networks for both bacteria and archaea were built based on 16S rRNA sequencing data. Two topological parameters, within-module connectivity (Zi) and among-module connectivity (Pi), were applied to identify keystone species within the molecular ecological networks. The threshold values for Zi and Pi were set at 2.5 and 0.62, respectively. Based on these values, network nodes (ASVs) were categorized into four types: peripheral nodes (Zi < 2.5, Pi ≤ 0.62), connectors (Zi < 2.5, Pi > 0.62), module hubs (Zi ≥ 2.5, Pi < 0.62), and network hubs (Zi > 2.5, Pi > 0.62) (Zhou et al., 2011). In ecological studies, peripheral nodes typically represent taxa with limited interactions within the network. Connector nodes are highly connected across different modules. Module hubs refer to nodes with strong connectivity within their own modules, while network hubs are nodes that exhibit high connectivity both within and between modules, thus playing a central role in the entire network structure (Berry and Widder, 2014).

### 2.6 Functional prediction-based analysis of carbon, nitrogen, and sulfur metabolic differences between bacteria and archaea

Microbial metabolic potential across sediment samples from different depths was predicted using the PICRUSt2 tool (Douglas et al., 2019), with functional assignments based on the Kyoto Encyclopedia of Genes and Genomes (KEGG) database (Kanehisa et al., 2023). The relative abundances of target functional modules derived from these predictions were specifically quantified. This approach provided hierarchical functional information ranging from pathways to genes and inference-based analysis of metabolic pathway species composition. More importantly, by leveraging the KEGG ortholog (KO) data obtained from the predictions, we specifically quantified the abundances of key functional genes involved in C, N, and S metabolism. This refined gene-level functional analysis aimed to elucidate the relative contributions and functional differentiation strategies of bacterial and archaeal communities in mediating the elemental cycling processes in deep-sea sediments.

## 3 Results and discussion

### 3.1 Diversity of bacterial and archaeal communities along sediment depth

**Figure 2:**
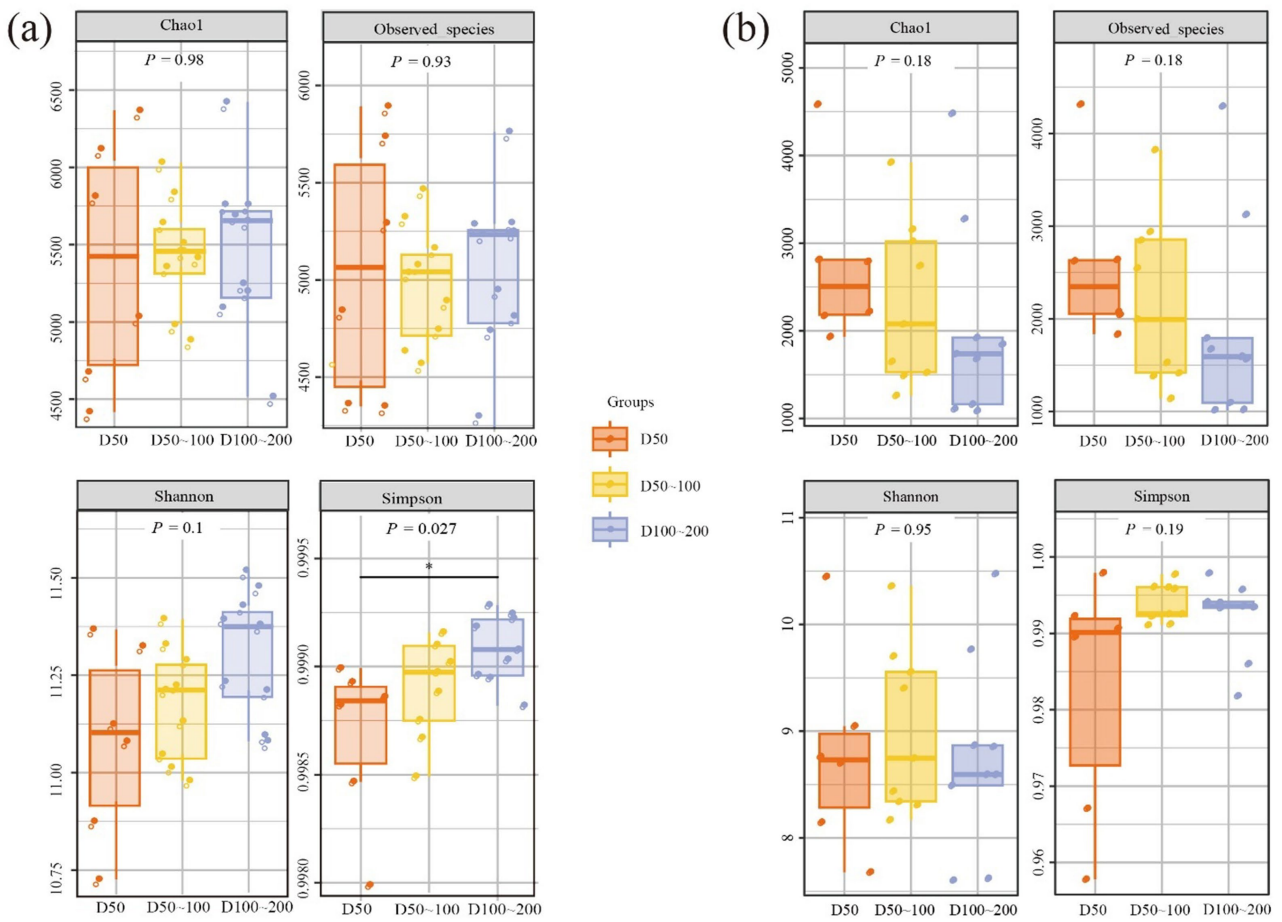
Bacterial and archaeal community diversity in ECS sediments based on 16S rRNA. **(a)** Chao1, Observed_species, Shannon, and Simpson, for bacterial community based on three stratifications. **(b)** Chao1, Observed_species, Shannon, and Simpson, for archaeal community based on three stratifications. *p*-values for the overall difference between groups obtained by the Kruskal-Wallis nonparametric test, as well as markers for the significance level of the difference obtained by the Dunn’s test *post hoc* two-by-two comparisons between groups are shown (*p < 0.05; **p < 0.01; ***p < 0.001).

The diversities and community compositions of bacterial and archaeal communities in ECS sediments were assessed via high-throughput sequencing of 16S rRNA gene amplicons, getting a rarefied ASV abundance table. Figures 2a,b depict vertical variations in species diversity indices. For bacteria, both the Chao1 and Observed species indices yielded *p*-values > 0.05 across depth layers. Although the Shannon index uncovered no significant differences in diversity among depths, the Simpson index exhibited clear variations. This disparity suggests that while species richness and evenness are comparable across depths, the identity and dominance of the most abundant taxa shift significantly, indicating shifts in dominant taxa across depths (Morris et al., 2014). Specifically, at 50 m, light-adapted taxa likely dominate due to high surface irradiance, reducing evenness. At greater depths (100–200 m), where conditions transition to low-light or aphotic environments, heterotrophic taxa become more prevalent, contributing to a more balanced community structure (Figure 2a) (Azam and Malfatti, 2007).

The archaeal community displayed markedly lower richness (1,000–4,000 ASVs) compared to bacteria, with greater variability observed in the mid-depth group (D50-100) (Figure 2b). The Shannon index significantly lower than bacterial values, yet no significant differences were detected among depth groups (*p* = 0.95), suggesting that archaeal diversity is overall lower but compositionally consistent across the investigated depth gradient. Simpson index values approached 1, indicating highly even community structures across depths. These results demonstrate a divergent response to depth gradients: bacterial diversity metrics vary, whereas archaeal diversity remains stable and even, suggesting fundamentally different strategies for occupying these environments.

### 3.2 Environmental and geographic drivers of bacterial and archaeal communities

**Figure 3:**
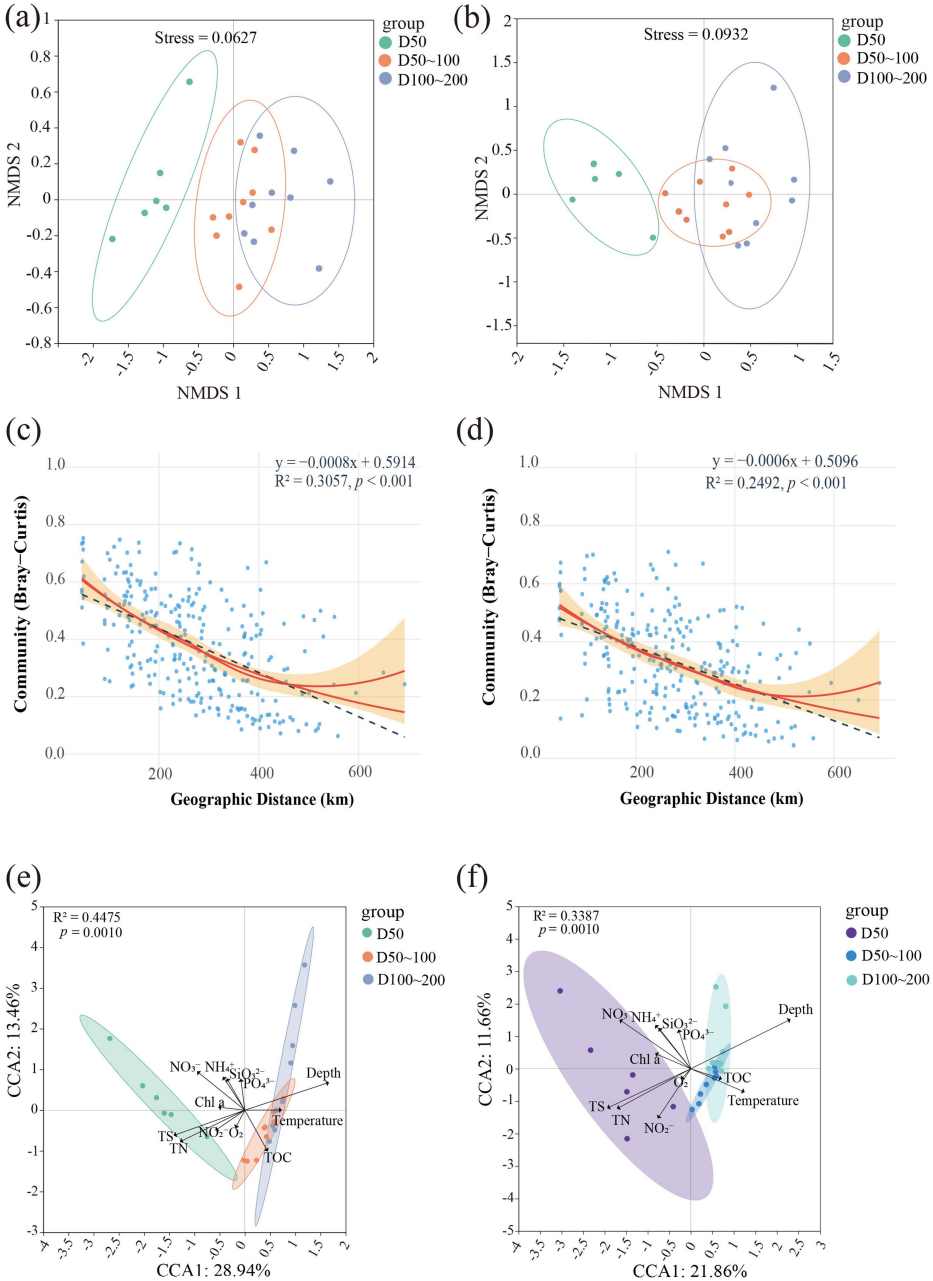
Vertical stratification and environmental drivers of bacterial and archaeal communities in East China Sea sediments. **(a)** Non-metric multidimensional scaling (NMDS) ordination of bacterial communities across depth gradients (D50, D50–100, D100–200 m). **(b)** NMDS ordination of archaeal communities across depth gradients. **(c)** Distance–decay relationship for bacterial communities based on Bray–Curtis dissimilarity and geographic distance (km). **(d)** Distance–decay relationship for archaeal communities. **(e)** Canonical correspondence analysis (CCA) of bacterial community structure constrained by environmental variables. **(f)** CCA of archaeal community structure constrained by environmental variables.

Non-metric multidimensional scaling (NMDS) was performed to further investigate the environmental drivers of these divergent strategies. The results revealed significant vertical stratification in both archaeal and bacterial communities across depth gradients (D50, D50-100, D100-200), which was associated with taxon-specific distribution patterns (Figures 3a,b). Bacterial communities (Figure 3a) exhibited distinct separation between surface (D50) and deeper sediments (D100-200), suggesting a strong response to depth-related environmental gradients (Hanson et al., 2012; Zinger et al., 2011). Conversely, although a degree of compositional overlap was visually observed in archaeal communities across depth gradients (Figure 3b), the magnitude of inter-depth community segregation for archaea was not statistically different from that for bacteria. This finding demonstrates that both archaeal and bacterial communities exhibit comparably complex distribution patterns across the sediment profile. Geographic distance-decay analysis showed varying patterns between bacteria and archaea (Figures 3c,d), which suggest a potential difference in their spatial assembly mechanisms. However, the subtle distinction observed warrants further investigation with a larger sample size. The distance-decay relationship was slightly steeper for bacterial communities than for archaea. While this trend suggests a potential for stronger dispersal limitation in bacteria, the small difference in slopes indicates that geographic distance play a similarly modest role in structuring both bacterial and archaeal communities in this system, with environmental filtering likely being the dominant assembly process for both. Canonical correspondence analysis (CCA) further identified key environmental drivers shaping community structures. For bacteria, the first two axes explained 44.75% of community variation (Figure 3e), whereas for archaea they explained 33.87% (Figure 3f). Bacterial communities were strongly influenced by total organic carbon (TOC), temperature, and depth, while archaea were more sensitive to oxygen availability and nutrient levels (Ramette and Tiedje, 2007). These results indicate that bacterial assemblages exhibit higher environmental responsiveness, whereas archaeal communities are relatively stable but constrained by oxygen and nutrient gradients (Nemergut et al., 2013).

### 3.3 Community structure of bacteria and archaea in East China Sea sediments

**Figure 4:**
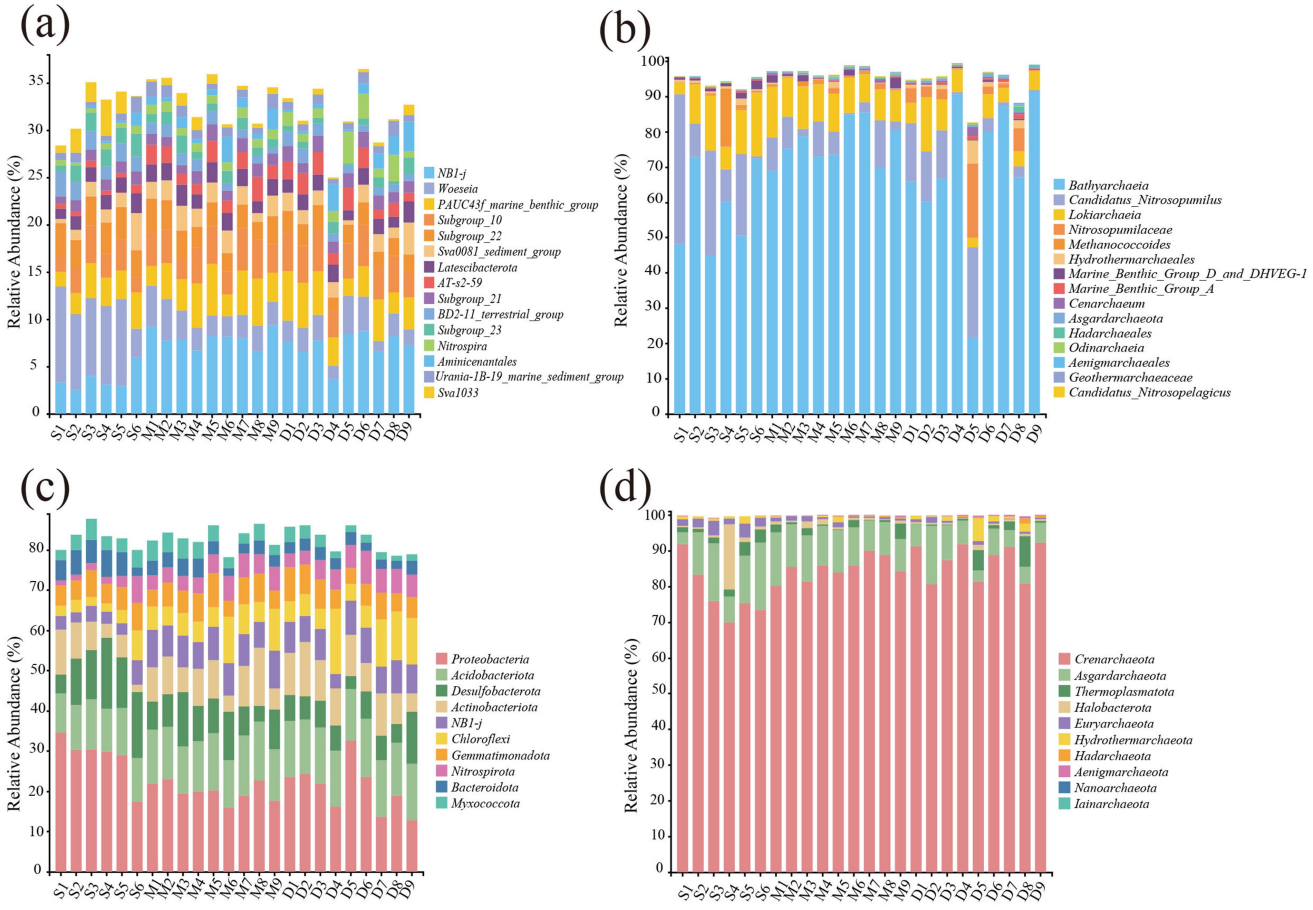
The distribution of bacteria **(a)** and archaea **(b)** at the genus level and the distribution of bacteria **(c)** and archaea **(d)** at the phylum level in East China Sea sediments.

The bacterial and archaeal communities were analyzed at both genus and phylum levels (Figure 4). A total of 15 dominant genera and 10 major phyla were identified. At the genus level, the dominant genus in shallow marine sediments is *Woeseia*, which transitions to *NB1-J* with increasing depth (Figure 4a) (Hoffmann et al., 2020). *Nitrospira*, a key nitrite-oxidizing genus, also increased at depth, consistent with its capacity for anaerobic respiration under reduced oxygen (Daims et al., 2015). In contrast, *Latescibacterota* displayed peak abundance in the D50-100 layer but declined sharply below 100 m, likely due to reduced nutrient inputs and changing redox conditions (Youssef et al., 2015). Archaeal communities (Figure 4b) were dominated by *Bathyarchaeia*, consistently abundant across all depths but especially enriched in D50-100 layers (Zhou et al., 2018). Shallow sediments (D50) also supported ammonia-oxidizing archaea such as *Candidatus_Nitrosopumilus* (Qin et al., 2014), while deeper sediments favored groups like *Marine_Benthic_Group_A* and *Marine_Benthic_Group_D*, likely linked to anaerobic organic matter degradation. Other archaeal taxa, including *Geothermarchaeaceae*, were rare, suggesting stricter environmental requirements (Kozubal et al., 2013).

At the phylum level (Figures 4c,d), bacterial communities exhibited higher taxonomic diversity, dominated by Proteobacteria, followed by Bacteroidota, Chloroflexi, and Desulfobacterota, whereas archaeal communities were dominated by Crenarchaeota (>70%) with low vertical variability. This contrast indicates complementary ecological roles: bacteria exploit a broad range of metabolic niches across depth gradients, whereas archaea specialize in energy-efficient pathways (e.g., methane cycling, ammonia oxidation) under reduced conditions (Parada and Fuhrman, 2017).

### 3.4 Co-occurrence patterns and keystone taxa in bacterial and archaeal networks

**Figure 5:**
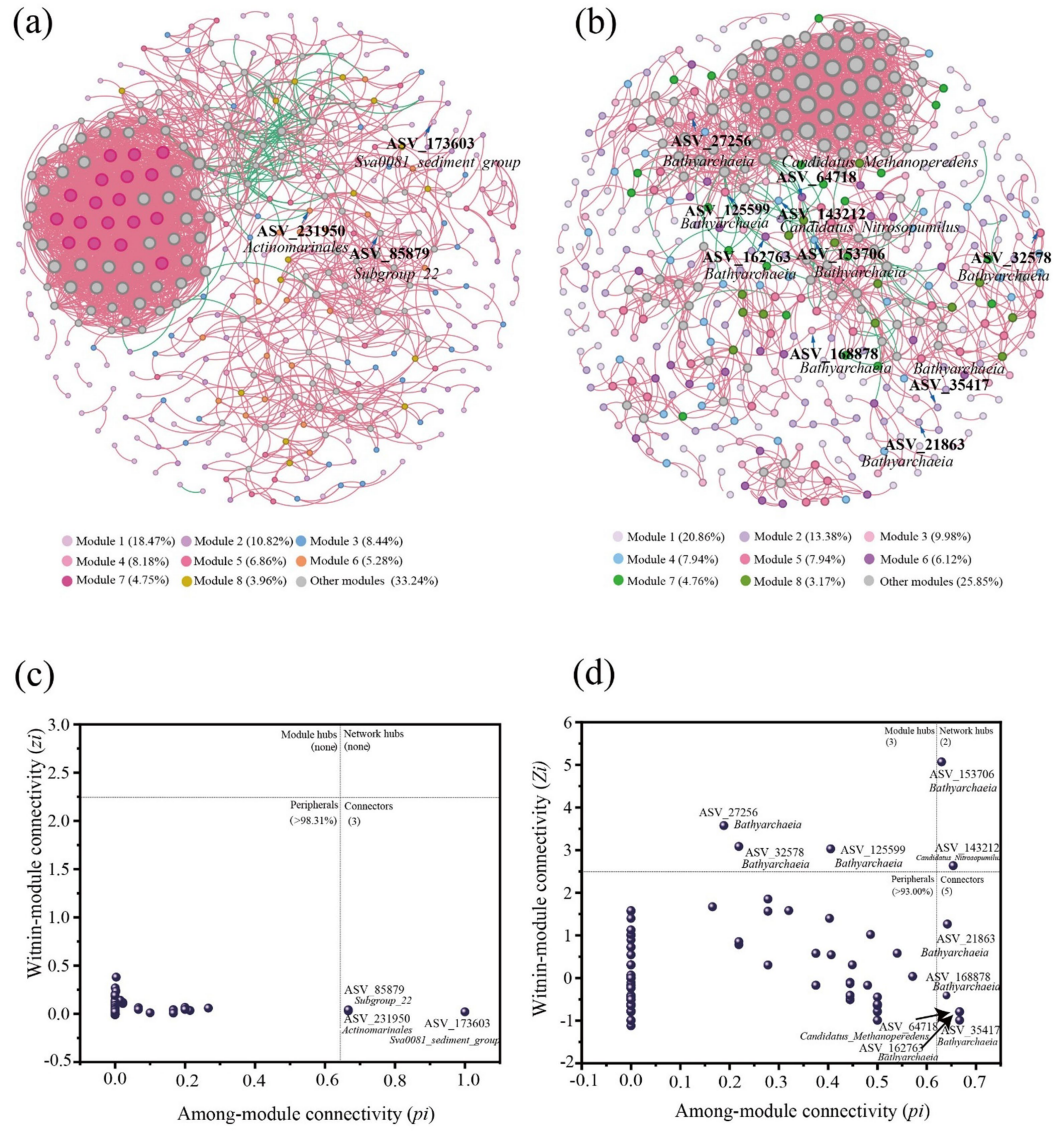
Interspecies ecological networks within bacteria **(a)** and within archaea **(b)** in ECS sediments. The Zi-Pi plot showing the distribution of bacterial ASVs **(c)** and archaeal ASVs **(d)** based on their module-based topological roles.

Microbial co-occurrence networks were constructed to explore the interactions and potential keystone taxa within bacterial and archaeal communities. As shown in Figure 5, both bacterial and archaeal networks exhibited pronounced modular structures. In the bacterial co-occurrence network (Figure 5a). Modules 1 and 2 comprised the largest proportions of the network, indicating that these bacterial groups played dominant roles. Certain core taxa, such as *Sva0081_sediment_group*, *Subgroup_22*, and members of *Actinobacteria*, displayed high within-module connectivity (Zi) (Figure 5c), highlighting their critical functions within specific modules. Over 98.31% of nodes were classified as peripheral, suggesting that bacterial communities were primarily composed of functionally specialized groups. Three taxa, *Subgroup_22*, *Actinomarinales*, and *Swd0031_sediment_group*, acted as connectors, exhibiting high among-module connectivity and serving as key bridges particularly between module 1 (18.47%) and module 2 (10.82%). Notably, no module hubs (Zi ≥ 2.5, Pi < 0.62) or network hubs (Zi > 2.5, Pi > 0.62) were detected, indicating a lack of central regulatory taxa in the bacterial network. This implies that bacterial community functioning rely on distributed cooperation among taxa rather than centralized control (Fuhrman et al., 2015). In contrast, the archaeal co-occurrence network (Figure 5b) was highly modular and overwhelmingly dominated by *Bathyarchaeia*. Forming two major functional units centered on module 1 (20.86%) and module 2 (13.38%) (Figure 5d). Representative examples include ASV_27256 in module 1 and ASV_32578 in module 2, both of which displayed strong within-module connectivity and likely coordinated key anaerobic metabolic processes. Archaeal networks exhibited moderately higher among-module connectivity, as several ASVs reached Pi values up to ≈ 0.6, mainly affiliated with *Bathyarchaeia*. While most archaeal ASVs still maintained relatively low Pi values, these highly connected *Bathyarchaeia* members acted as potential connectors across modules, linking distinct ecological functions, such as methanogenesis in surface sediments (module 1) and anaerobic methane oxidation in deeper layers (module 2) (Haroon et al., 2013). This partially coupled network structure, driven by *Bathyarchaeia*, enhance the functional resilience of archaeal communities under dynamic chemical gradients (Lazar et al., 2016; Evans et al., 2019).

In summary, the bacterial network shows clear functional separation, with most modules interacting only weakly with each other. In contrast, the archaeal network forms tightly clustered groups, but a few *Bathyarchaeia* members act as important connectors, linking different modules and functions. These differences suggest that bacteria and archaea adopt distinct survival strategies in marine sediments: bacteria are more specialized and occupy separate niches, while archaea are more flexible and better adapt to changing environmental conditions.

### 3.5 Differential responses of bacterial and archaeal communities to environmental variables

**Figure 6:**
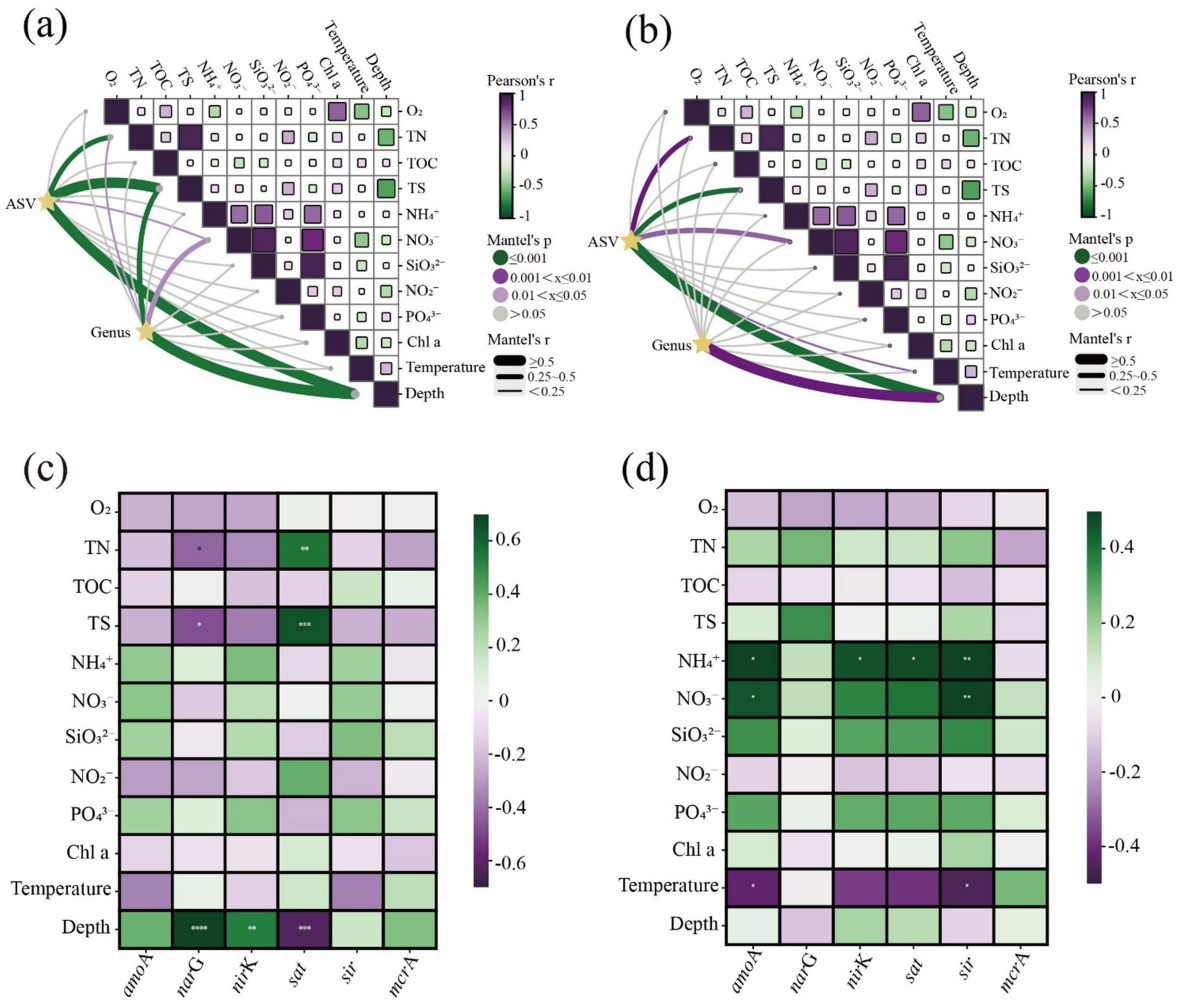
Influence of spatial and biogeochemical factors on bacterial **(a)** and archaeal **(b)** communities in ECS sediments. Correlation patterns of nitrogen and sulfur cycling genes with nutrient factors in bacteria **(c)** and archaea **(d)**.

To comprehensively understand microbial community responses to environmental factors, Mantel tests were performed at ASV and genus levels to assess bacterial/archaeal environmental associations (Figure 6). Depth emerged as a dominant factor, exerting significant influence on both bacterial (Figure 6a) and archaeal (Figure 6b) communities at both taxonomic levels. This supports the notion that vertical stratification in the water column plays a universal role in shaping prokaryotic assemblages through redox gradients and pressure changes (Sunagawa et al., 2015).

At the ASV level, the bacterial communities showed a strong positive correlation with total sulfur (TS), highlighting sulfur cycling in shaping bacterial community structures (Muyzer and Stams, 2008). With increasing depth, sulfate-reducing bacteria such as *Desulfatiglanis* thrive under low-oxygen conditions by using sulfate as a terminal electron acceptor to anaerobically degrade organic substrates (Rabus and Wilkes, 2020), promoting sulfur reduction, sulfide accumulation, and subsequent effects on benthic redox state and microbial succession. Therefore, TS act as a key driver, promoting the transition of bacterial communities toward anaerobic functional groups like *Desulfatiglanis*, reflecting ecological shifts in deeper sediment layers (Canfield et al., 1993). Functional gene analyses (Figures 6c,d) further supported these patterns: bacterial genes such as *nar*G (nitrate reductase), *nir*K (nitrite reductase), *sat* (Serine Acetyltransferase), and *amo*A (ammonia monooxygenase) were strongly correlated with TOC and NH_4_^+^, suggesting their crucial roles in denitrification, nitrification, and sulfate reduction. For archaea, the *amo*A gene was significantly associated with NH₄^+^ and depth, highlighting their dominance in ammonia oxidation, while other archaeal pathways such as nitrate reduction and sulfur metabolism showed limited correlations. Interestingly, *mcr*A genes exhibited slightly higher associations in deeper sediments, implying a potential archaeal contribution to anaerobic methane cycling. Archaeal communities were less influenced by TS. At the genus level, archaea were less affected by depth than bacteria, and their correlations with NO_3_^−^ and TS were negligible. This suggests a higher level of adaptability to low-oxygen, deep, and energy-limited environments among archaeal taxa (Orsi et al., 2020).

Correlations also existed among environmental variables themselves. We conducted linear analyses of significantly correlated environmental variables identified in the Mantel test (Supplementary Figure S1). Strong positive correlations were observed between TN and TS (Supplementary Figure S1a), PO_4_^3−^ and SiO_3_^2−^ (Supplementary Figure S1b), NO₃^−^ and PO_4_^3−^ (Supplementary Figure S1c), and NO_3_^−^ and SiO_3_^2−^ (Supplementary Figure S1d), with Spearman *ρ* values exceeding 0.8 (*p* < 0.001). These findings indicate that these nutrients share similar sources or be co-regulated by common environmental factors. The strong TN-TS correlation likely reflects their coupled release during organic matter input and decomposition, particularly under hypoxic or anaerobic sedimentary conditions where nitrogen and sulfur cycles are often microbially mediated. The covariation of PO_4_^3−^ and SiO_3_^2−^ indicate synchronized uptake by diatoms and other phytoplankton with a concurrent demand for phosphorus and silicon (Brzezinski, 1985). The high correlations between NO_3_^−^ and both PO_4_^3−^ and SiO_3_^2−^ suggest co-input from external pollution sources such as agricultural runoff or domestic sewage (Howarth et al., 2011), and also be influenced by shifts in phytoplankton community composition.

Collectively, these results underscore the distinct yet interconnected roles of bacterial and archaeal assemblages in regulating sedimentary biogeochemical cycles. Bacteria dominate diverse pathways related to organic matter decomposition, nitrogen transformation, and sulfur cycling, whereas archaea are primarily responsible for ammonia oxidation and contribute to methane cycling in deeper layers. Building on these insights, the next section focuses on how these microbial functions are linked to environmental gradients and potential interdomain interactions within the sediment ecosystem.

### 3.6 Depth-related patterns in functional annotation of carbon, nitrogen, and sulfur metabolism pathways

**Figure 7:**
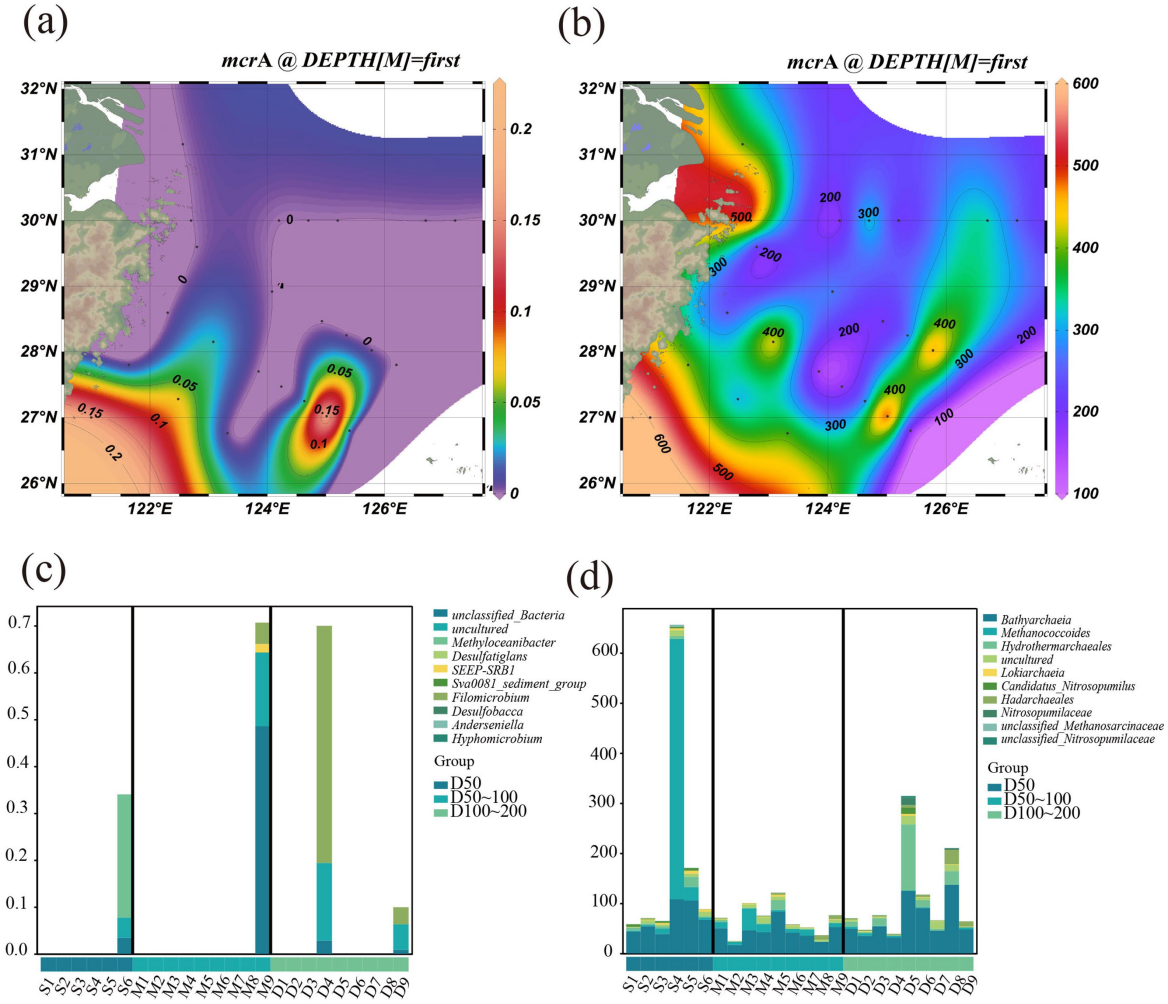
*mcrA* in bacteria **(a)** and archaea **(b)** and species composition of methanogenic metabolic pathways in bacteria **(c)** and archaea **(d)** at the genus level.

To further elucidate microbially driven biogeochemical cycles in coastal sediments, we predicted the abundance of key functional genes involved in carbon (C), nitrogen (N), and sulfur (S) metabolism, and analyzed their spatial distribution using heatmap analysis, which revealed distinct functional differentiation between bacterial and archaeal communities across the three elemental cycles. For the C cycle, archaea are primarily characterized by their involvement in methanogenesis, with *mcr*A being the key functional gene (Evans et al., 2019). The spatial distribution of the *mcr*A gene, revealed clear heterogeneity along the East China Sea shelf sediments (Figure 7). In Figure 7a, the relative abundance of *mcr*A exhibited pronounced hotspots (>0.15) around 27°N and 29°N, while offshore regions displayed much lower levels, indicating localized enhancement of methane-related activity. These findings align with studies reporting similar spatial patterns in methane-cycling microbial communities in marine environments (Yao et al., 2025). Figure 7b shows that these hotspots were primarily concentrated within the 300–500 m depth range, suggesting that methane metabolism is particularly active in the mid-depth transition zones, likely linked to higher organic matter availability and reducing conditions. Figures 7c,d highlight a distinct compositional divergence between bacterial and archaeal communities involved in methane cycling across depth gradients. For bacterial communities (Figure 7c), shallow (L50) and deep (L200) sediments were dominated by unclassified taxa, *Methylomicrobacter*, *Deep-sea SRB1*, and other sulfate-reducing bacteria (SRB). In contrast, while bacteria play a major role in organic carbon oxidation, their capacity for anaerobic carbon metabolism is relatively limited. Given that deep marine zones are often low-oxygen or anoxic, archaea utilizing CO₂ and H₂ as substrates for methanogenesis demonstrate greater adaptive capability and metabolic flexibility (Hinrichs and Boetius, 2003).

By contrast, the archaeal communities (Figure 7d) exhibited clear depth dependency. In the shallow layer (L50), *Bathyarchaeia* and *Methanococcales* dominated, reflecting a preference for organic-rich, reducing environments that favor methane production. At greater depths (L200), the dominance shifted toward *Methanomicrobiales* and unclassified *Nitrosopumilaceae*, suggesting that archaea play a central role in methanogenesis and anaerobic methane oxidation under oxygen-depleted, oligotrophic conditions.

**Figure 8:**
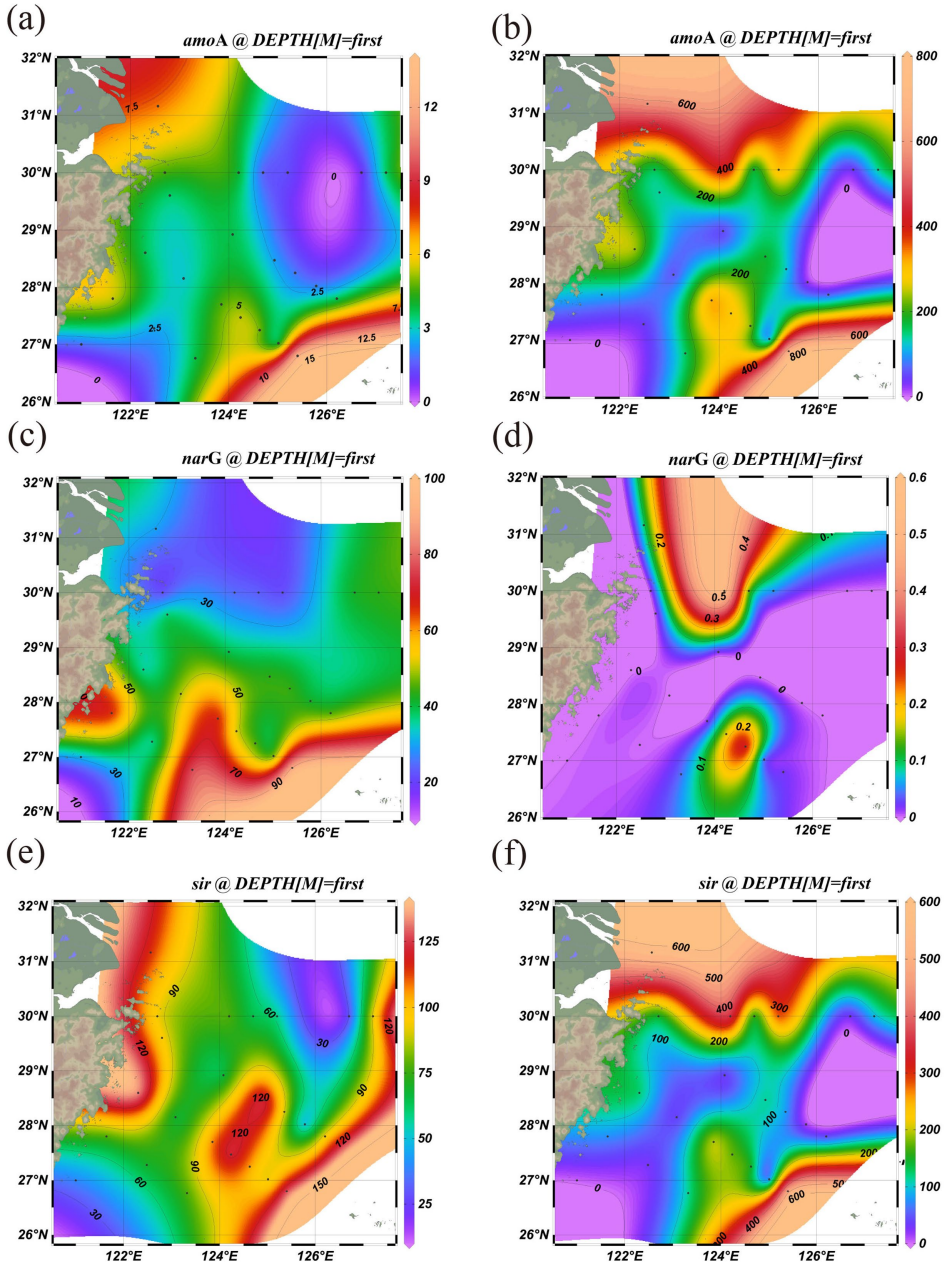
Potential nitrogen cycling related pathways functional bacteria activity via KEGG orthology: *amoA* in bacterial **(a)** and archaeal **(b)**, *narG* in bacterial **(c)** archaeal **(d)**, *sir* in bacterial **(e)** and archaeal **(f)**.

For the nitrogen cycle (Figures 8a–d), bacterial genes such as *amo*A and *nar*G were broadly detected at the DNA level, especially in sediments. This indicates that bacteria have the potential for nitrification (oxidizing NH_4_^+^ to NO_3_^−^) and possess strong capacity in oxidative nitrogen cycling (Bouskill et al., 2012). Additionally, the high abundance of bacterial *nir*K (Supplementary Figure S2a) suggests active participation in denitrification, converting NO_2_^−^ to gaseous nitrogen (N₂). Archaeal *amo*A and *nar*G genes were also widely distributed and maintained high abundance even in low-oxygen or anoxic zones, suggesting an important role in sediments ammonia oxidation and nitrate respiration. Although archaeal *nir*K (Supplementary Figure S2b) abundance was lower than that in bacteria, it still showed high expression in specific deep-sea areas (e.g., northern and offshore regions), indicating potential higher resilience to environmental stress (Qin et al., 2020). Overall, archaea demonstrate stronger ecological adaptability in anaerobic nitrogen metabolism, while bacteria are more active in the oxidative nitrogen cycle. In terms of the sulfur cycle (Figures 8e, f), bacterial genes *sir* (sulfite reductase) was significantly more abundant in sediments than in archaea, suggesting bacterial dominance in sulfate activation and early-stage sulfate reduction (Müller et al., 2015). Bacteria appear to rely more heavily on oxidative sulfur species as electron acceptors, thus exhibiting greater advantage in surface layers. The heatmap revealed that *mcr*A gene abundance was significantly higher in certain regions for archaea than bacteria, indicating that archaea dominate methane production in these reductive environments. In contrast, archaea showed higher *sir* gene abundance in deeper, more reductive zones, indicating an adaptive advantage in reductive sulfur cycling, such as the reduction of sulfate to H₂S. A comprehensive characterization and analysis of the sat gene were also conducted (Supplementary Figures S2c,d). Archaea generally possess greater anaerobic metabolic capabilities and can maintain active sulfur metabolism pathways under low-oxygen conditions (Wagner et al., 2016). Moreover, the broader spatial distribution of archaeal sulfur metabolism genes suggests greater functional resilience across ecological niches.

**Figure 9:**
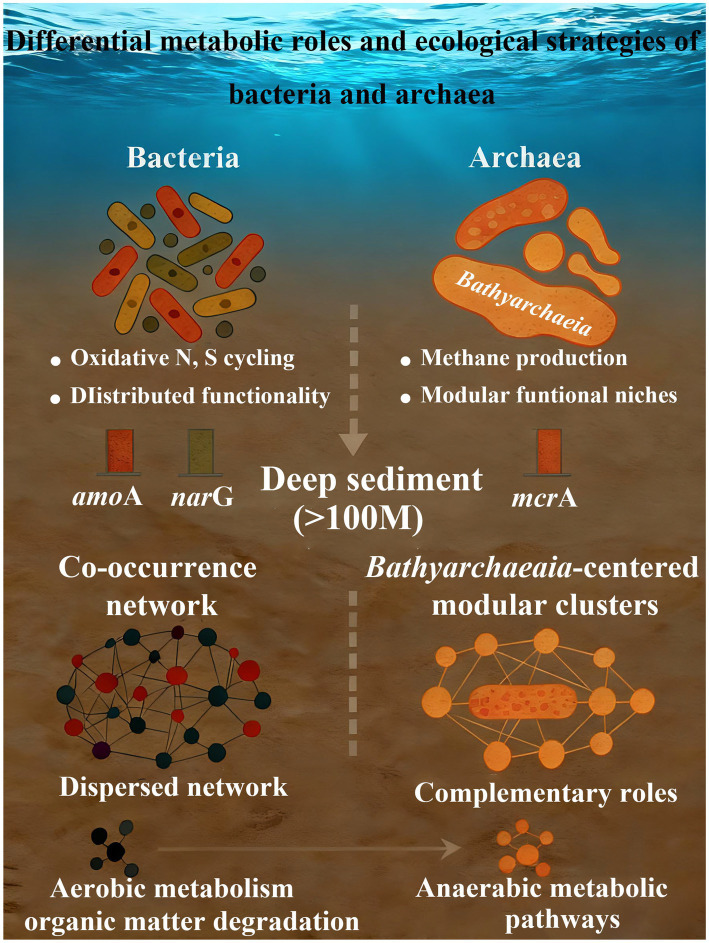
Differential metabolic roles and ecological strategies of bacteria and archaea.

In summary, bacterial communities exhibit broader and more efficient metabolic capacities under surface and oxidative conditions, whereas archaea demonstrate specialized metabolic strategies and ecological advantages under extreme, low-oxygen, or highly reductive environments (Figure 9). The complementary roles facilitate cycling in marine ecosystems.

## 4 Conclusion

This study comprehensive assessed the vertical distribution patterns, ecological strategies, and functional potentials of bacterial and archaeal communities in ECS sediments, clarifying core mechanisms of microbially mediated elemental cycling in continental shelf environments. Bacterial communities exhibited high metabolic plasticity, supporting niche diversification and predominance in oxidative nitrogen and sulfur cycling, with their spatial distribution primarily shaped by local environmental gradients rather than geographic distance, while archaeal communities showed functional stability and spatial restriction, playing key roles in methanogenesis and anaerobic nitrogen/sulfur metabolism (especially in anoxic sediments below 100 m) driven by strong dispersal limitation and nutrient gradient sensitivity. Bacterial networks relied on distributed cooperation among specialized taxa, whereas archaeal networks formed highly modular “functional islands” dominated by *Bathyarchaeia*. Functional predictions further underscored the complementary contributions of bacteria and archaea to carbon, nitrogen, and sulfur biogeochemical cycles.

Through integrated taxonomic-ecological-functional analysis, this study explicitly illustrated the core mechanism of microbially mediated elemental cycling in continental shelf sediments: bacteria drive oxidative nitrogen and sulfur transformations through distributed functional division, while archaea participate in carbon degradation and methanogenesis via modular pathways. This functional complementarity highlighted the coupled nature of carbon-nitrogen-sulfur (C-N-S) cycling in shelf sediments. The results provided key ecological evidence for elucidating the mechanisms by which shelf ecosystems buffer anthropogenic nutrient inputs, mitigate greenhouse gas emissions, and respond to climate-driven marine changes.

## Data Availability

The data presented in this study are publicly available. The data can be found at: https://www.ncbi.nlm.nih.gov, accession numbers PRJNA1309059 and PRJNA1309283.

## Footnotes

1
https://www.chiplot.online/
