# Supplementary material for: Bacteria-archaea metabolic complementarity as a driver of ecosystem functioning in Chinese coastal sediments
Source: Front Microbiol. 2026 Feb 17;17:1785657. doi: 10.3389/fmicb.2026.1785657 (PMC12954776; doi:10.3389/fmicb.2026.1785657)
Supplement: Supplementary file 1 [file Table_1.DOCX]

**Bacteria–Archaea Metabolic Complementarity as a Driver of Ecosystem Functioning in Chinese Coastal Sediments**

Xi Yuan ^a, b^, Xiao-Lin Liu ^c^, Si-Qi Ye ^a, b^, Shou-Qing Ni ^d^, Zhi-Bin Wang ^b, a*^

^a^ *School of Life Sciences, Shandong University, Qingdao**, Shandong, 266237, China*

*^b^ Shenzhen Research Institute of Shandong University, Shenzhen, Guangdong, 518000, China*

*^c^* *Institute of Marine Science and Technology, Shandong University, Qingdao, Shandong, 266237, China.*

*d* *School of Environmental Science and Engineering,* *Shandong University,* *Qingdao, Shandong,* *266237, China*

^*^ *Corresponding author:* [zbwang@sdu.edu.cn](mailto:zbwang@sdu.edu.cn)

**Table**

Table S1 Detailed information of each site.

**Figures**

Fig. S1. Inter-factor correlation analysis of environmental variables

Fig. S2. Potential nitrogen cycling related pathways functional bacteria activity via KEGG orthology

**Table S1 Detailed information of each site.**

| **Station** | **Longitude**  **(E)** | **Latitude**  **(N)** | **O₂**  **(µmol/dm³)** | **NH₄⁺**  **(µmol/L)** | **NO₃⁻**  **(µmol/L)** | **SiO₃²⁻**  **(µmol/L)** | **NO₂⁻**  **(µmol/L)** | **PO₄³⁻**  **(µmol/L)** | **Chl a**  **(mg/m3)** | **Temperature (℃)** | **Depth (m)** |
| --- | --- | --- | --- | --- | --- | --- | --- | --- | --- | --- | --- |
| S1 | 122.56 | 31.16 | 449.4830412 | 2.01 | 8.97 | 17.64 | 0.46 | 0.99 | 0.77 | 12.8437 | 20 |
| S2 | 122.7 | 30 | 432.5667055 | 1.29 | 7.73 | 15.94 | 0.42 | 0.83 | 0.04 | 13.9941 | 35 |
| S3 | 122.8 | 29.6 | 375.9669895 | 1.6 | 3.58 | 9.06 | 0.34 | 0.43 | 0.11 | 16.8067 | 50 |
| S4 | 122.3 | 28.6 | 403.3345917 | 1.31 | 2.6 | 8.15 | 1.46 | 0.61 | 0.25 | 18.1363 | 46 |
| S5 | 121.64 | 27.8 | 439.6318474 | 1.45 | 5.81 | 13.15 | 0.75 | 0.73 | 0.11 | 16.0373 | 33 |
| S6 | 121 | 27 | 404.77048 | 1.29 | 3.61 | 10.71 | 1.04 | 0.66 | 0.42 | 17.158 | 42 |
| M1 | 124.2 | 30 | 484.644445 | 0.9 | 5.12 | 14.14 | 0.45 | 0.87 | 0.06 | 12.8377 | 61 |
| M2 | 124.7 | 30 | 430.3664389 | 1.17 | 1.51 | 7.27 | 1.76 | 0.56 | 0.28 | 14.9637 | 56 |
| M3 | 125.2 | 30 | 447.9542845 | 0 | 0 | 0 | 0 | 0 | 0.16 | 14.8917 | 57 |
| M4 | 124.08 | 28.92 | 421.3653481 | 1.46 | 5.22 | 17.21 | 0.63 | 1.01 | 0.04 | 17.3019 | 75 |
| M5 | 124.93 | 28.47 | 597.4174642 | 0.95 | 1.46 | 5.15 | 0.49 | 0.55 | 0.97 | 15.4414 | 99 |
| M6 | 123.08 | 28.15 | 407.0350401 | 1.52 | 1.72 | 6.58 | 0.69 | 0.42 | 0.13 | 18.7106 | 80 |
| M7 | 123.85 | 27.7 | 399.1983761 | 1.56 | 3.07 | 10.37 | 0.3 | 0.59 | 0.04 | 18.0936 | 92 |
| M8 | 124.24 | 27.47 | 362.6653775 | 1.65 | 3.61 | 12.11 | 0.32 | 0.69 | 0.05 | 18.0317 | 80 |
| M9 | 122.48 | 27.28 | 395.0978792 | 0 | 0 | 0 | 0 | 0 | 0.06 | 18.4334 | 98 |
| D1 | 126.7 | 30 | 382.5535019 | 1.43 | 2.11 | 5.95 | 0.25 | 0.41 | 0.03 | 15.2712 | 95 |
| D2 | 127.2 | 30 | 369.1590216 | 1.27 | 2.86 | 9.8 | 0.25 | 0.48 | 0.06 | 15.7114 | 102 |
| D3 | 125.35 | 28.25 | 424.0156693 | 0.92 | 5.54 | 15.26 | 0.68 | 1.14 | 0.05 | 16.1533 | 110 |
| D4 | 125.78 | 28.02 | 438.2316777 | 1.23 | 4.4 | 15.9 | 0.47 | 0.92 | 0.05 | 16.8213 | 107 |
| D5 | 126.2 | 27.8 | 381.8534171 | 1.84 | 4.86 | 14.6 | 0.41 | 0.94 | 0.02 | 15.6527 | 178 |
| D6 | 124.63 | 27.25 | 361.1294771 | 1.4 | 3.68 | 10.97 | 0.32 | 0.82 | 0.05 | 18.3905 | 101 |
| D7 | 125.01 | 27.02 | 444.4395729 | 1.01 | 3.45 | 11.33 | 0.32 | 0.69 | 0.07 | 16.9918 | 107 |
| D8 | 125.4 | 26.8 | 378.1458249 | 1.66 | 4.61 | 12.59 | 0.28 | 0.89 | 0.38 | 16.6157 | 148 |
| D9 | 123.32 | 26.76 | 390.4616031 | 1.43 | 3.88 | 13.26 | 0.66 | 0.82 | 0.09 | 17.7778 | 143 |


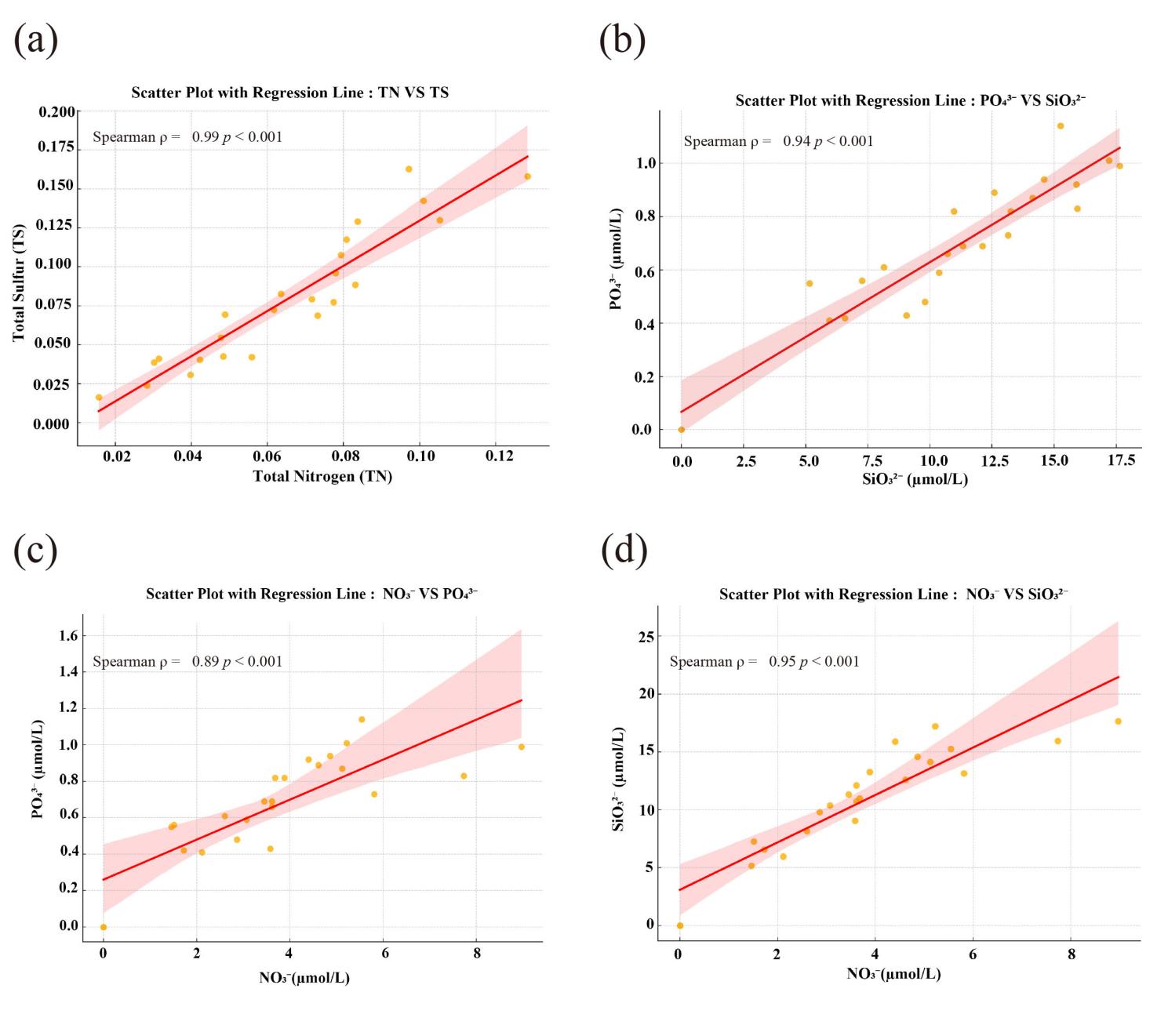


**Fig. S1. (a)-(d) Inter-factor correlation analysis of environmental variables**


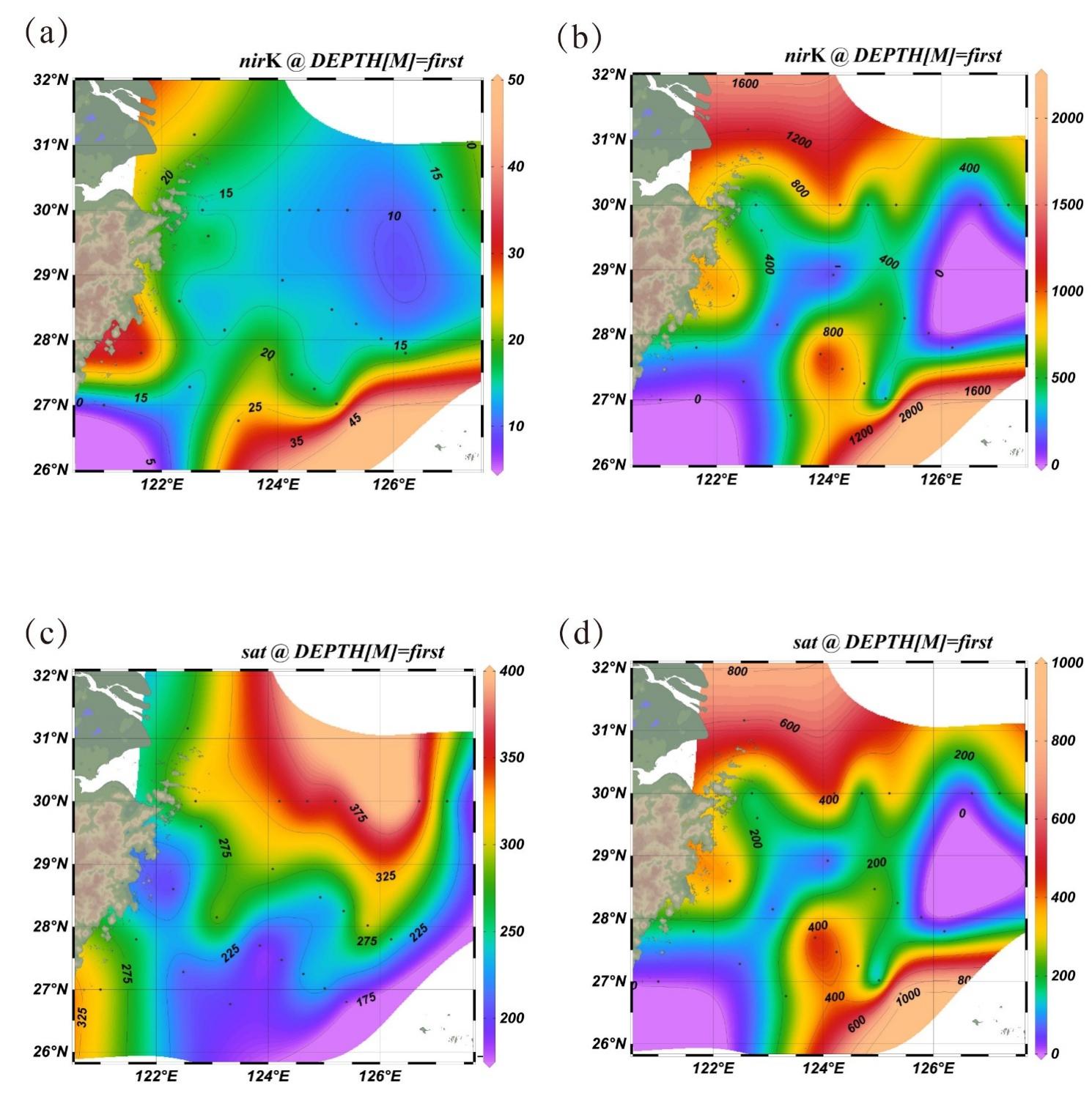


**Fig. S2. Potential nitrogen cycling related pathways functional bacteria activity via KEGG orthology: *nir*K in bacterial (a) and archaeal (b), *sat* in bacterial (c) archaeal (d).**
